# Should Opticians Practise Medicine?1Read by title at the meeting of the Central New York Medical Association, Rochester, October 20, 1896.

**Published:** 1897-05

**Authors:** Alvin A. Hubbell

**Affiliations:** Buffalo, N. Y.; Professor of ophthalmology and otology in the Medical Department of Niagara University, 212 Franklin Street


### SHOULD OPTICIANS PRACTISE MEDICINE?[1]

By ALVIN A. HUBBELL, M. D., Buffalo, N. Y.,
Professor of ophthalmology and otology in the Medical Department of Niagara University.

TO PRACTISE medicine is to give one's time, more or less, to the examination and treatment of deformities, diseases or injuries. The limitation or narrowing of these to a certain class of abnormalities or to certain organs does not change the nature of the practice. When an optician "tests" the eyes, he does what the physician does in any abnormal condition : he examines the case. When he prescribes glasses, he does what the physician does: he makes a diagnosis and prescribes a remedy, and often with the avowed purpose of relieving headaches or other diseases.

Outside of presbyopia (old-age sight), every case requiring glasses has some condition of the eyes that is wrong. If it is a refractive error, there is, essentially, a deformity. If it is impaired vision, there is something abnormal either in the refraction or in the structures of the eye. If it is headache or other neurosis, this is in itself a disease. Whoever undertakes to adjust glasses, therefore, is dealing with abnormal conditions, and is seeking to relieve distress. This is all there is to the practice of medicine, and this is what the "refracting" optician is engaged in.

1. Read by title at the meeting of the Central New York Medical Association, Rochester, October 20, 1896.

Those abnormalities of the eye and their reflexes which suggest glasses as a remedy are most varied and complex. To determine their nature and trace their relations can be done only by one versed in physics, anatomy, physiology and pathology, and even then obstacles are often met with which are well-nigh insurmountable. A diagnosis being arrived at, the proper remedy can only be suggested by one whose therapeutics include any agency applicable to the ascertained condition. This may be glasses, or it may be hygiene, or it may be medicine, or it may be an operation.

Indeed, the subjects of refractive errors and muscular defects, in their whole range, both in examination and correction, are medical in the most technical and profound sense. No subject in medicine has been so diligently studied, and has yielded such fruitful results, as these; but physicians, and physicians alone, have done the work. The attempt to single out these conditions, and set them apart as a little kingdom by themselves, is irrational and absolutely without justification.

The optician who is not a physician may have a good knowledge of optics and lenses; but the eye is more than an optical instrument with fixed lenses and mathematical curves and surfaces, to be readjusted or neutralised. It is a living organ, subject to many diseases itself, and so intimately related to the system at large that its conditions not only affect other parts by reflex action, but it, in turn, is affected by influences and diseases originating in other organs. Only the physician can understand these and deal with them properly. The optician may have some notions of the local anatomy, physiology and pathology of the eye, but the vagueness and limitation in which he holds them render them useless, or perhaps dangerous, if he undertakes to apply them. Not possessing the requisite knowledge, he may fail to recognise a disease of the eye which, if neglected, may lead to blindness. He may not discover symptoms in the eye which, if seen by a physician, would reveal the presence of a remote or constitutional disease, the early treatment of which would save the individual's life. If he prescribes, he may not fully meet the requirements of the case, as other therapeutic measures may be needed besides glasses. Indeed, he may aggravate reflexes, cause disease, foster delay and otherwise do positive harm.

The optician has his legitimate field as a mechanic, manufacturer and tradesman. He bears the same relation to the public as the druggist or instrument-maker. Any examining or prescribing which he does is not founded on reliable knowledge, and any claims which he makes to fitness for such are mere pretense. The practice which he seeks being medical, the same requirements should prevail here as in any other department of medicine, and he should be made amenable to the same laws regulating it. The purpose of our medical laws is to protect the public from the practices of the incompetent. Let these laws, then, be made to reach and stay this growing abuse of "testing" or "refracting" eyes by those who are not qualified to do so. The requisite for the practice of any department or specialty in medicine is a knowledge of the whole body of medicine, and the practice of so-called "optometry" should be no exception.

212 Franklin Street.

